# An implantable pump Lenus pro® in the treatment of pulmonary arterial hypertension with intravenous treprostinil

**DOI:** 10.1186/s12890-017-0474-7

**Published:** 2017-12-02

**Authors:** Marcin Kurzyna, Katarzyna Małaczyńska-Rajpold, Andrzej Koteja, Agnieszka Pawlak, Łukasz Chrzanowski, Michał Furdal, Zbigniew Gąsior, Wojciech Jacheć, Bożena Sobkowicz, Justyna Norwa, Tatiana Mularek-Kubzdela, Adam Torbicki

**Affiliations:** 1 Department of Pulmonary Circulation, Thromboembolic Diseases and Cardiology, Centre of Postgraduate Medical Education, European Health Centre Otwock, Borowa 14/18, 05-400 Otwock, Poland; 2 1st Department of Cardiology, University of Medical Sciences, Poznan, Poland; 3 Department of Anesthesiology and Intensive Care, European Health Centre Otwock, Otwock, Poland; 4 Department of Invasive Cardiology, Central Clinical Hospital of the Ministry of the Interior and Administration, Warsaw, Poland; 5 Department of Cardiology, Medical University of Lodz, Lodz, Poland; 6 Department of Cardiology, Provincial Specialist Hospital in Wroclaw, Research and Development Centre, Wrocław, Poland; 7 Department of Cardiology, SHS, Medical University of Silesia, Katowice, Poland; 8 2nd Department of Cardiology, School of Medicine with Dentistry Division, Medical University of Silesia, Zabrze, Poland; 9 Department of Cardiology, Medical University of Bialystok, Białystok, Poland

**Keywords:** Pulmonary arterial hypertension, Treprostinil, Implantable pump, Quality of life, SF-36 form

## Abstract

**Background:**

Subcutaneous treprostinil is a prostacyclin analogue used to treat pulmonary arterial hypertension (PAH). Due to local pain it can cause a deterioration of heart related quality of life (HRQoL) or even abandonment of treatment. The aim of this paper was to assess the feasibility of treatment with intravenous treprostinil administered by means of the Lenus Pro® implantable pump.

**Methods:**

This was a retrospective, multi-center study involving 12 patients (8 females) with PAH treated with a subcutaneous infusion of treprostinil with intolerable pain at the infusion site. Clinical evaluation, including HRQoL assessment with SF-36 questionnaire was performed, before pump implantation and 2–9 months after. The median time of follow-up time was 14 months (4–29 months).

**Results:**

After implantation of the Lenus Pro® pump, no statistically significant changes were observed in the 6-min walking distance and NT-proBNP. After implantation 50% of patients were in II WHO functional class (33% before, *p* = 0,59). There was a significant improvement in HRQoL within the Physical Component Score (28 ± 7 vs 38 ± 8 pts., *p* < 0,001) and in specific domains of SF-36 form: physical role (31 ± 7 pts. vs. 41 ± 12 pts., *p* = 0,03), bodily pain (31 ± 12 vs. 50 ± 14 pts., *p* = 0,02), and vitality (37 ± 8 pts. vs. 50 ± 14 pts., *p* = 0,03). During the periprocedural period, one patient developed a recurrent haematoma at the implantation site. During follow-up in one patient, the drug delivering cannula slipped out of the subclavian vein, what required repositioning repeated twice, and in another patient an unexpected increase in the drug administration rate was observed.

**Conclusions:**

In patients with PAH who do not tolerate subcutaneous infusion of treprostinil, the use of the Lenus Pro® implantable pump results in significant subjective improvement of vitality and physical aspect of the HRQoL with acceptable safety profile.

## Background

Treprostinil, a prostacyclin analogue, is a drug that is widely used to treat pulmonary arterial hypertension (PAH) [1–3]. Its efficacy was confirmed in studies that compared it to placebo [1, 4] and to epoprostenol [4]. Due to the stability of treprostinil sodium solution and its relatively long half-life compared to prostacyclin [5, 6], the drug enabled PAH patients to receive safe long-term treatment. Treprostinil is administered as a continuous subcutaneous infusion using an insulin-like pump. In the case of this route of administration, its half-life is about 3 h [6]. Unfortunately, due to reaction at the infusion site many patients report significant deterioration of quality of life, and some of them (about 5–10%) even abandon treatment [1, 7]. There are trials in progress to find a more convenient method of administration for this drug. The efficacy of oral administration has been uncertain – reports are contradictory [8–12], while inhalation remains a valid alternative for patients in a less advanced stage of the disease [13]. Therefore, for patients whose illness is more severe, only continuous parenteral administration of the drug remains an option.

The Lenus Pro® implantable pump appears to be a promising alternative to an external pump. By means of this method, treprostinil sodium is administered as a continuous intravenous infusion, and the drug reservoir is refilled every 28 days. Thermal stability of treprostinil at body temperature was confirmed during a 60-day observation [5]; concentrations of the drug administered intravenously are comparable to subcutaneous administration, and the only differing parameter is a shorter half-life of less than 1 h [14]. The first experiences with implantable pumps originate in Austria [15, 16] and Germany [17] and present this method of treatment as a milestone in PAH therapy. In Poland, the first implantation of a Lenus Pro® pump took place in 2013 [18]. Functionally similar pump, was tested in US with comparable results in efficacy and safety [19].

The aim of this paper was to assess the feasibility of treatment with intravenous treprostinil administered by means of the Lenus Pro® implantable pump and its impact at quality of life.

## Methods

During the years from 2013 to 2016 the number of patients with PAH treated with subcutaneous infusion of treprostinil sodium (Remodulin®, PubChem CID: 23663413) in Poland ranged from 52 to 98. Among them 12 patients (8 males/4 females, aged 42 ± 13 yrs) treated with subcutaneous treprostinil infusion were qualified for implantable pump because they did not tolerate the therapy due to local inflammatory reactions, pain at the infusion site and infection-related complications. Additional inclusion criteria were good clinical response for sc treprostinil infusion guarantying improvement during further therapy. We retrospectively reviewed their clinical data and HRQoL (Short Form 36, SF36) [20, 21] questionnaires obtained before and during 2–9 months after Lenus Pro® pump implantation. Non-invasive clinical evaluation was made, including: WHO functional class (WHO FC), 6-min walking test (6MWT), and concentration of NT-proBNP. All patients gave informed consent for pump implantation. Local Bioethics Committee at Poznan University of Medical Sciences was notified about the study and decided this retrospective analysis does not require formal approval (KB 576/17).

### Lenus pro implantable pump

**Fig. 1:**
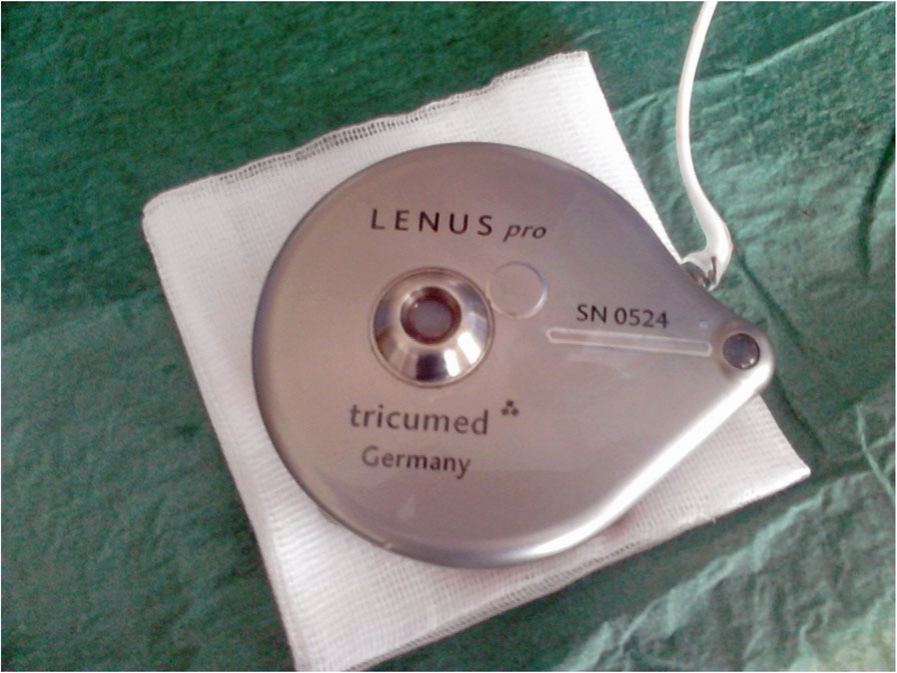
Photography of the Lenus Pro® pump prepared for implantation

Lenus Pro® pump (Tricumed Medizintechnik GmbH, Kiel, Germany) is a metal device of diameter of 8 cm and a thickness of 2 cm with two compartments inside (Fig. 1). One with capacity of 40 ml is the drug reservoir and latter is filled with noble gas. Both compartments are divided by movable membrane and filling the pump with drug effects in gas compression and causes drug flow toward the patients. The special chip with thin capillary created inside of piece of glass causes constant drug flow (about 1,3 ml/day) irrespectively of gas pressure (high after filling and low close to next refill). As the flow of the drug solution is constant for specific device the dose of the drug is adjusted by changing its concentration. Pump should be refilled every about 28 days by percutaneous puncture of silicone port located on the top of the devices. The second smaller port is designed for pump flushing. No mechanical parts requiring powering are installed inside causing no need for periodic replacement like in case of pacemaker. The pump is usually located in subcostal area and connected with tunnelized catheter inserted via subclavian vein into superior caval vein. The Lenus Pro pump was approved in EU for use in humans according to the Medical Devices Act and is MRI compatible up to 3 Tesla machines.

### Pump implantation procedure

Prior to pump implantation the subcutaneous dose of treprostinil was escalated up to the highest dose tolerated by a particular patient. The procedure of pump implantation was carried out in European Health Center Otwock under general anesthesia (*n* = 11) or shallow analgosedation (fentanyl and propofol) combined with local anesthesia with lidocaine 2% (*n* = 1) in Zabrze. At first, a silicone catheter with a diameter of 6F was introduced via the subclavian vein into the superior vena cava using Seldinger’s technique. The tip of the catheter was placed in the superior vena cava, 1–2 cm above its ostium into the right atrium. Subsequently, the Lenus Pro® pump was implanted either in the right or left subcostal area suprafascially, and in one patient, subfascially due to very scarce subcutaneous adipose tissue. A catheter was then tunneled from the implanted pump under the skin of the abdomen and chest to the subclavian area where it was connected to the tip of the catheter introduced into the subclavian vein by means of a special connector. The pump was filled with treprostinil prior to its implantation, without changing the dose of the drug administered by subcutaneous infusion. The external pump delivering the drug subcutaneously was detached after about 3 h, when treprostinil administered by mean of the Lenus Pro® pump filled its catheter and reached the patient’s bloodstream. During this period patient stayed in intensive care unit.

### Statistical analysis

Data are presented as means and standard deviations (SD) or medians and interquartile range (IQR). The Wilcoxon signed rank test was used for statistical analysis of data obtained prior to and following implantation. A *p* value <0.05 was considered significant.

## Results

At the time of starting therapy with subcutaneous treprostinil, all patients exhibited symptoms of advanced PAH – mean pulmonary artery pressure was 64 ± 17 mmHg, cardiac index was 2.56 ± 1.1 L/min*m2, mean right atrial pressure 10 ± 4 mmHg, and pulmonary vascular resistance was 14.8 ± 7.1 Wood Units. 75% of patients were WHO functional class III, while the remaining ones were WHO functional class IV. The median time of subcutaneous therapy prior to Lenus Pro® pump implantation was 8 months (from 1 to 51 months, IQR 6–18).

The clinical characteristics of patients who had a Lenus Pro® pump implanted are presented in Table 1.

**Table 1:** Clinical characteristics of patients treated with subcutaneous treprostinil referred for the pump implantation and the postimplantation parameters

Pt	Age range [yrs.]	PAH etiology	Duration of sc. therapy [months]	Dose sc. [ng/kg/min]	Dose iv. [ng/kg/min]	WHO FC before implantation	WHO FC after implantation	FU [months]	Complications
1	42	Corrected CHD	6	38,0	40,0	II	II	29	None
2	28	Corrected CHD	50	62,5	80,0	III	III	24	None
3	37	Idiopathic PAH	6	20,0	30,5	III	III	21	Unexpected acceleration in drug delivery
4	40	Idiopathic PAH	8	22,5	22,5	II	II	19	Slight dislocation of pump without clinical implications
5	68	Connective tissue disease	8	27,0	30,0	II	III	12	Died because of lung cancer, no problems with the pump
6	54	Eisenmenger	6	25,5	43,5	III	II	16	None
7	25	Eisenmenger	9	41,3	42,0	III	III	15	None
8	38	Idiopathic PAH	27	40,2	41,3	III	III	13	Dislocation of the catheter, decrease in delivery rate
9	57	Connective tissue disease	10	33,9	35,4	II	II	11	None
10	53	Idiopathic PAH	29	40,4	44,0	II	II	10	None
11	30	Corrected CHD	7	17,0	40,0	III	IV	4	Recurrent hematoma at implantation site, decrease in delivery rate
12	32	Connective tissue disease	2	39,0	46,0	III	II	4	None

*PAH* – pulmonary arterial hypertension, *CHD* – congenital heart disease, *WHO FC* – WHO functional class, *FU* – follow-up [months]

### Efficacy of switch from sc to iv infusion with Lenus pro® pump

**Fig. 2:**
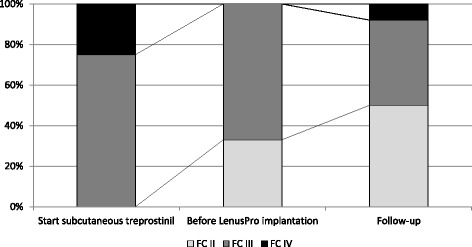
Changes in WHO functional class assessed at the time of starting subcutaneous treprostinil infusion, before Lenus Pro® pump implantation and in follow-up

**Fig. 3:**
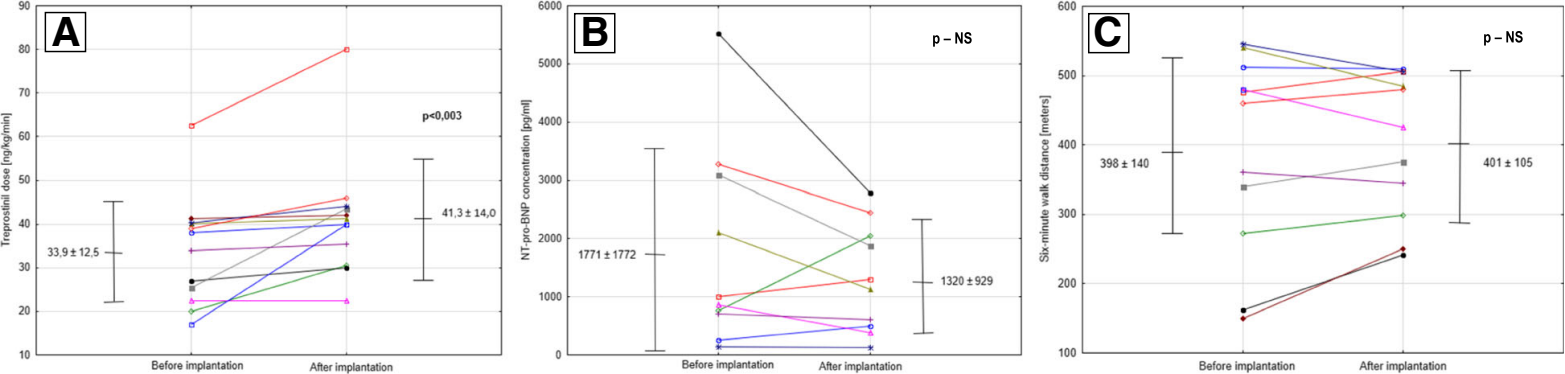
Changes in treprostinil dose (**a**), NT-proBNP concentration (**b**), 6MWT (**c**) treprostinil after implantation of Lenus Pro® pump

Immediately before Lenus Pro® pump implantation, 4 (33%) patients were functional class II and 8 (67%) were functional class III. After implantation, 6 (50%) patients were functional class II, and 5 (42%) and 1 (8%) were functional class III and IV, respectively (*p* = 0,59; Fig. 2). The treprostinil dose used was increased significantly after implantation of the Lenus Pro® pump – 33.9 ± 12.5 vs. 41.3 ± 14.0 ng/kg/min (*p* = 0.003). The blood concentration of NT-pro-BNP decreased from 1771 ± 1772 pg/ml to 1320 ± 929 pg/ml. However, this trend did not prove to be statistically significant (*p* = 0.24). Six-minute walking distance did not change – 398 ± 140 m vs. 402 ± 105 m (*p* = 0.59) (Fig. 3).

### Safety of implantable pump

During implantation, no complications associated with surgical anesthesia were observed. During the postoperative period, in 4 cases small hematoma in the pump implantation bed was diagnosed that required a single evacuation by puncture. In one patient (pt. #11), puncturing of the pump bed area was required 3 times due to recurrence of the hematoma. This patient presented coagulopathy because of splenomegaly associated with liver cirrhosis resulting in thrombocytopenia.

During the follow-up with the median time of 14 months (from 4 to 29 months, IQR 11–20), one patient (#5) died of lung cancer, while others survived and were treated further with treprostinil.

In one patient, the drug delivery cannula slipped out of the subclavian vein during stretching gymnastics. It required the repositioning catheter twice [22]. In two cases, there was a reduction in the drug administration rate that required the flushing procedure to be carried out (pts #8 and #11) resulted in restoration of the original flow ratio. Those patients reported more aggravated fatigue without signs of right ventricular decompensation. Flushing was performed through service point with heparinized saline after complete emptying of the pump. In one patient (pt #3), an unexpected increase in the drug administration rate was observed – from 1.3 mL/day to 1.7 mL/day, which resulted in total emptying of the pump 1 day before refill visit with symptoms of deterioration of exercise capacity and fatigue, but without hemodynamic collapse. The flow acceleration required adjustment of the dose and frequency of follow-up visits. Other problems with the implantable pumps were of local nature and did not require any additional interventions. No clinically significant complications were observed during pump refill procedures, which were performed in authors’ centers.

### Health related quality of life

With regard to quality of life evaluated by means of the SF36 form, there was a significant improvement within the Physical Component Score – 28 ± 7 vs. 38 ± 8 points. (*p* < 0.001) and in specific domains: Physical Role – 31 ± 7 vs. 41 ± 12 points, (*p* = 0.03), Bodily Pain – 31 ± 12 vs. 50 ± 14 points (*p* = 0.02), and Vitality – 37 ± 8 vs. 50 ± 14 points (*p* = 0.03; Fig. 3). At general question “How would you rate your health in general compared to period before pump implantation?” 10 patients (83%) answered “Much better now” and 2 patients answered “Somewhat better now” and “About the same”, respectively.

## Discussion

The most important finding emerging from this study is the confirmation of positive effect at patients’ HRQoL and relative safety of treating PAH with treprostinil by means of the Lenus Pro® subcutaneous implantable pump. Patients evaluated in the study benefited after implantation of the implantable pump despite the fact it was no change in active substance but only the route of administration. It may partially result from the fact that it was possible to increase the medication dose after pump implantation thanks to elimination of the infusion site pain. Although the concentration of NT-proBNP did not change significantly and the 6-min walking distance did not increase, patients reported better physical performance, which was consistent with functional class evaluation made by a physician. However, assessment of functional class was not blinded and was based on patients’ report, so placebo effect cannot be completely excluded. An increase in the 6MWT distance was noticeable in some patients – particularly in those, in whom this distance at the baseline was very short (about 150 m) (Fig. 3b). Improvement in treatment outcomes may also have been caused by elimination of activities related to external pump handling, which had resulted in unintentional errors. Moreover, better outcomes could also be caused by improvement of patient compliance in terms of continuous drug administration and maintenance of a fixed or increasing dose. It seems that due to implantation of a subcutaneous pump, the therapeutic effect of treprostinil has taken advantage to the fullest extent – without limitations associated with the previous method of administration.

**Fig. 4:**
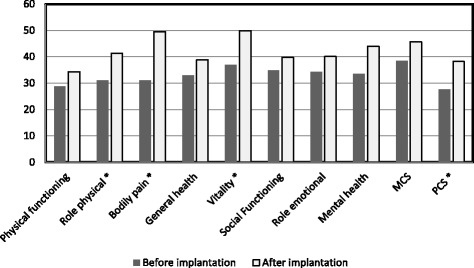
Changes in quality of life measured with the SF-36 ver2 form. Norm based scores provided for 9 patients who completed the SF36 form. MCS – mental component score PCS – physical component score, * - *p* < 0.05

From the SF-36 questionnaire, it was found that improvement in HRQoL resulting from pump implantation was related first and foremost to reduction of experienced pain and the physical limitations associated both with the disease and the presence of an external pump. Bourge et al. also found improvement in activity scale of CAMPHOR questionnaire after switch from external system for delivery of treprostinil to implantable pump of different type [19]. Pain at the infusion site results from the topical effect of treprostinil that dilates the vessels in the subcutaneous tissue, leading to its hyperemia and pain fibers irritation [13] . Administrating the drug directly into the central vein allows the patient to avoid that bothersome side effect. Additionally, the presence of an external pump is associated with limitations to physical activity resulting from the patient being afraid of damaging the delicate infusion set construction and the drug-delivering catheter attached to it, and the necessity to detach the pump e.g. when bathing. Additionally, an external pump requires the drug to be replenished every 2–4 days, and in the case of some patients this requires assistance of a third person due to manual or language limitations. The complexity of treprostinil therapy by an external pump is therefore much higher than in the case of an implantable pump. Although the procedure of pump implantation itself bears a certain risk, the subsequent handling, which requires only a monthly refilling in the treating center, is much more patient-friendly. In this study, this directly translated into an improvement of the physical aspect of the quality of life (PCS – physical component score) (Fig. 4). Interestingly, patients demonstrated an increase in vitality after implantation of the Lenus Pro® pump, as reported in appropriate domain of the SF-36 questionnaire. However, due to a lack of objective indicators of clinical improvement in the form of 6MWT and NT-proBNP, it can be assumed that the increase in vitality results from “rebuilding” the energy that patients lost due to the use of an external pump and due to the feeling of stigmatization associated with it. Additional factor leading to improve in the vitality might be withdrawal some analgesics taken previously because of pain at infusion site.

This study showed safety of surgical procedure of pump implantation with general anesthesia in severely ill PAH patients. In Poland treatment with treprostinil is the second line anti-PAH therapy, restricted for patients deteriorating on oral therapies or being in functional class IV at diagnosis. In periprocedural period only minor complications were observed including hematoma in the pump implantation bed that required an evacuation by a puncture. In one case recurrent hematoma was found in patient with coagulopathy but it was also managed without surgical intervention. Richter et al. in series of 51 patients who were implanted with Lenus Pro pump reported two deaths because of right heart failure during hospital stay. Three patients suffered from atrial fibrillation related to catheter irritation, and in one case hematoma and one case of seroma with catheter dislocation occurred [23].

Major complications during follow-up included an unexpected increase in the drug flow rate in the pump that required a change in the drug concentration and frequency of refilling, and dislocation of the drug-delivering catheter that required two repositioning procedures. The issue of drug flow changes requires further in-depth analysis and verification of the filling protocol, as it might be potentially life threatening complications related to stop of drug delivery for several hours. In few available publications, the that complications have not been described [15, 16]. However, in 2013 producer of Lenus Pro pump issued safety note informing that more than 10% acceleration in drug delivery might happen, especially when pumps are used for longer periods of time (2 to 4 years). It was postulated that the treprostinil would cauterize the glass surface within the capillary increasing its diameter. The corrective action was introduced by the new development of a chip canal with a more resistant glass [24]. As the pumps implanted in our patients came from upgraded series it should not be responsible for delivery acceleration. The fever or direct exposition of abdomen for sun which may increase the gas pressure inside pump and accelerate delivery rate were either excluded in this case. The issue of instability of flow delivery was also reported by Bourge et al. with different type of implantable pump [19].

The catheter dislocation was the next important complication during follow-up. In observed patient, it was clearly dependent on excessive physical activity including stretching and rap dancing. After reposition and cessation of above mentioned activities the catheter’s position was stable. Thus, it seems sensible to carry out periodic follow-ups of the catheter location using an imaging technique (e.g. chest X-ray or fluoroscopy).

Our study confirmed safety of refiling process as well. Hohenforst-Schmidt et al. described one case, where erroneous administration of a monthly dose of treprostinil outside of the lumen of the pump while refilling resulted in circulatory collapse that was managed within a day using supportive therapy [25].

### Limitations of the study

The main limitation of the study was a small number of patients and a lack of the control group. When considering the number of pumps implanted in relation to the number of patients treated with subcutaneous treprostinil, it seems that only 5–10% of patients will require changing of the subcutaneous therapy. It is consisted with percentage of patients withdrawing from sc therapy observed in previous trials [1, 2]. The lack of the control group may make it difficult to evaluate the impact of the placebo effect on improvement of the HRQoL. However, we found no negative effect on NT-pro-BNP, an objective indicator of heart failure. Moreover, this study presents only short-term and mid-term effects of changing the way of treprostinil administration. In order to carry out long-term evaluation, it would be necessary to prolong the observation period.

## Conclusions

In patients with PAH who do not tolerate subcutaneous treprostinil infusion, the use of the Lenus Pro® implantable pump eliminates pain at infusion site and results in significant subjective improvement of vitality and physical aspect of the HRQoL. Therapy with the use of the Lenus Pro® pump is characterized by efficacy comparable to that of subcutaneous administration of treprostinil and acceptable safety profile, which however requires further observation.

## Abbreviations

6MWT: 6-mintute walking test
CHD: congenital heart disease
FU: follow-up [months]
HRQoL: health-related quality of life
NT-proBNP: N-terminated brain natriuretic propeptide
PAH: pulmonary arterial hypertension
PCS: physical component score
WHO FC: World Health Organization functional class
